# Ovarian Cell Encapsulation in an Enzymatically Crosslinked Silk-Based Hydrogel with Tunable Mechanical Properties

**DOI:** 10.3390/gels7030138

**Published:** 2021-09-10

**Authors:** Hafez Jafari, Arezoo Dadashzadeh, Saeid Moghassemi, Payam Zahedi, Christiani A. Amorim, Amin Shavandi

**Affiliations:** 1 BioMatter Unit, Ecole Polytechnique de Bruxelles, Université Libre de Bruxelles, B-1050 Brussels, Belgium; seyed.hafez.jafari@ulb.be; 2 Pole de Recherche en Gynecologie, Institut de Recherche Experimentale et Clinique, Université Catholique de Louvain, B-1200 Brussels, Belgium; arezoo.dadashzadeh@uclouvain.be (A.D.); saeid.moghassemi@uclouvain.be (S.M.); 3 Nano-Biopolymers Research Laboratory, School of Chemical Engineering, College of Engineering, University of Tehran, Tehran 1417613131, Iran; phdzahedi@ut.ac.ir

**Keywords:** ovarian stromal cells encapsulation, injectable hydrogel, enzymatic crosslinking, artificial ovary, silk fibroin

## Abstract

An artificial ovary is a promising approach for preserving fertility in prepubertal girls and women who cannot undergo current cryopreservation strategies. However, this approach is in its infancy, due to the possible challenges of creating a suitable 3D matrix for encapsulating ovarian follicles and stromal cells. To maintain the ovarian stromal cell viability and proliferation, as a first step towards developing an artificial ovary, in this study, a double network hydrogel with a high water swelling capacity (swelling index 15–19) was developed, based on phenol conjugated chitosan (Cs-Ph) and silk fibroin (SF) through an enzymatic crosslinking method using horseradish peroxidase. The addition of SF (1%) to Cs (1%) decreased the storage modulus (G’) from 3500 Pa (Cs1) to 1600 Pa (Cs-SF1), and the hydrogels with a rapid gelation kinetic produced a spatially homogeneous distribution of ovarian cells that demonstrated 167% proliferation after 7 days. This new Cs-SF hydrogel benefits from the toughness and flexibility of SF, and phenolic chemistry could provide the potential microstructure for encapsulating human ovarian stromal cells.

## 1. Introduction

The transplantation of cryopreserved ovarian tissue is one of the main strategies for restoring the fertility of cancerous patients. However, this approach is not recommended for patients that have been diagnosed with some tumors, such as leukemia, neuroblastoma, and Burkitt lymphoma, because of the possibility of reintroducing the malignant cells present in the ovarian tissue fragments after grafting [1]. An option for these patients can be developing a 3D transplantable artificial ovary that embeds ovarian cells and preantral follicles from frozen-thawed ovarian tissue [2,3]. To this end, a 3D scaffold should be developed to encapsulate and preserve the spherical architecture of follicles, which is essential to maintain cell–cell and cell–matrix interactions, and to achieve folliculogenesis resumption [4,5]. The hydrogels used for ovary tissue engineering must be biocompatible and must resemble the normal ovary in terms of biomechanical and biological properties. Moreover, hydrogels should be biodegradable, to support follicle development from 30 µm in diameter (primordial stage) to 20 mm (preovulatory stage) [6,7].

Various biopolymers, such as chitosan (Cs), hyaluronic acid, alginate, gelatin, and silk fibroin (SF), have been used to develop injectable hydrogels for cell therapy and cell encapsulation applications [8]. Among them, Cs is a promising candidate due to its biocompatibility, biodegradability, and inherent biological activity, such as antibacterial, antioxidant, and anti-inflammatory activities [9]. However, Cs-based hydrogels suffer from some critical limitations, such as poor water solubility, poor mechanical properties, and low viscoelasticity, which hamper its application for cell encapsulation [10].

Enzymatic crosslinking methods using horseradish peroxidase (HRP) enzyme for developing Cs-based hydrogel have been widely used, due to HRP’s mild reaction condition in physiological environments, tunable gelation time, and mechanical properties [11]. Phenolated Cs (Cs-Ph) can readily form a hydrogel using horseradish peroxidase (HRP) and hydrogen peroxide (H_2_O_2_) as crosslinking coagents [11].

Several biopolymers, such as alginate, hyaluronic acid, gelatin, and SF, can be incorporated into the hydrogel to address the low viscoelasticity, toughness, and resilience capability of a Cs-based hydrogels formula [12,13]. SF forms an elastic/biocompatible hydrogel via enzymatic crosslinking, using HRP and H_2_O_2_, without requiring the conjugation of phenol (Ph) groups that is required for enzymatic crosslinking. Indeed, Ph groups of tyrosine residues on the SF chains are oxidized in the presence of H_2_O_2_, and HRP leads to the formation of covalent dityrosine crosslinks [14]. The addition of SF to Cs hydrogel can increase the toughness, viscoelasticity, and mechanical performance of the hydrogel, promoting cell behaviors such as the cell proliferation and migration required for tissue regeneration [15].

In this study, we synthesize a hydrogel using Cs and SF using an enzymatic crosslinking method, to evaluate its ability to maintain human ovarian stromal cell viability and proliferation, as a first step towards developing an artificial ovary. Cs was conjugated with Ph groups, enabling it to be crosslinked in the presence of HRP and H_2_O_2_. A series of hydrogels made of Cs, SF, and their mixture (Cs-SF) were developed, and the effect of the polymers’ concentrations on the gelation time, and the rheological properties of the hydrogels, were investigated. Moreover, the impact of different concentrations of SF in Cs-SF hydrogel on the microstructure, swelling index, and degradation behavior of the hydrogel was evaluated. Finally, the effect of the hydrogel composition on the proliferation and viability of encapsulated ovarian stromal cells was investigated.

## 2. Results and Discussion

### 2.1. Cs-Ph Synthesis

**Figure 1:**
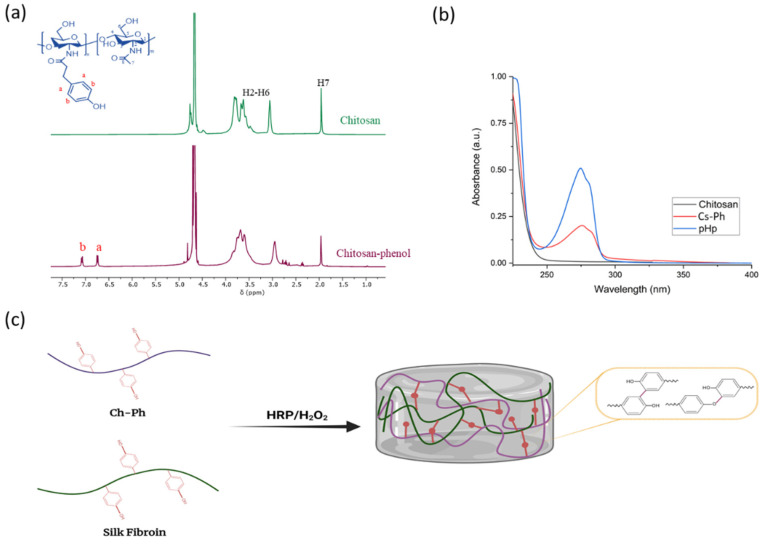
(**a**) ^1^H NMR spectra of the Cs-Ph conjugate and unmodified Cs; (**b**) UV-vis spectra of Cs-Ph, unmodified Cs, and pHp; (**c**) Schematic illustration of Cs-SF hydrogel through an enzyme-mediated crosslinking method using HRP and hydrogen peroxide (H_2_O_2_).

The synthesis route of Ch-Ph is shown in Figure 1c. An N-hydroxy succinimide/1-ethyl-3-(3-dimethylaminopropyl) carbodiimide (EDC/NHS)-mediated amide coupling reaction was used to introduce Ph groups into the Cs backbone. 3-(4-hydroxyphenyl) propionic acid (pHp) was activated by EDC to form an active O-Acylisourea intermediate that was susceptible to react with the primary amino groups of Cs, resulting in the conjugation of the Ph groups on the Cs chains. In general, the introduction of hydrophilic moieties into the Cs backbone improves the water solubility of the Cs by converting amine groups to glycolamide groups and disrupting the intermolecular hydrogen, which can facilitate the biomedical application of Cs [16].

^1^H NMR and UV-vis spectrometry analyses were carried out to evaluate the modification and incorporation of the Ph groups into the Cs backbone. The Cs ^1^H NMR spectra (Figure 1a) showed characteristic peaks at δ = 2.0 ppm (corresponding to the three protons of the *N*-acetyl glucosamine (GlcNAc) unit) and δ = 3.0 ppm (corresponding to the H-2 proton of glucosamine (GlcN) residues [17]), and approximately δ = 4.7 ppm of the Cs glucopyranose ring C∓H overlaps with the water peak [17]. The Cs-Ph spectrum shows the same characteristic peaks, in addition to two peaks at δ = 6.6 ppm and δ = 7.1 ppm, corresponding to the aromatic protons. These data confirm the incorporation of phenolic moieties on the CS backbone [18].

Moreover, the UV spectra of Cs, Cs-Ph, and pHp (Figure 1b) showed that a new peak was observed on the Cs-Ph spectra, indicating the presence of the aromatic groups on the Cs backbone, confirming the successful conjugation of pHp to the Cs. Additionally, the substitution degree of the Ph groups was determined to be 139 μmol/g of polymer, using the calibration curve of pHp.

### 2.2. Hydrogel Formation and Gelation Time

The incorporation of phenolic compounds in the Cs backbone and the tyrosine groups of SF allows for the development of hydrogels through enzymatic crosslinking. Cs-Ph and silk fibroin hydrogels were then formed through enzymatic crosslinking. The hydrogel formation was induced by mixing, in the first instance, the polymers with HRP, and then by adding H_2_O_2_. Horseradish peroxidase activated by H_2_O_2_ catalyzes the oxidation of the phenolic groups in the Cs and SF chains, generating two phenoxy radicals in one catalytic cycle. The phenoxy radicals can, subsequently, react together through a radical coupling reaction, resulting in the formation of C∓C and C∓O bonds [19]. The phenolic moieties at the ends of the polymers (e.g., pHp for Cs and tyrosine for SF) are coupled to each other via a C∓C bond or a C∓O bond between the carbon atom at the ortho position of Ph groups and the phenolic oxygen (Figure 1c) [18].

**Figure 2:**
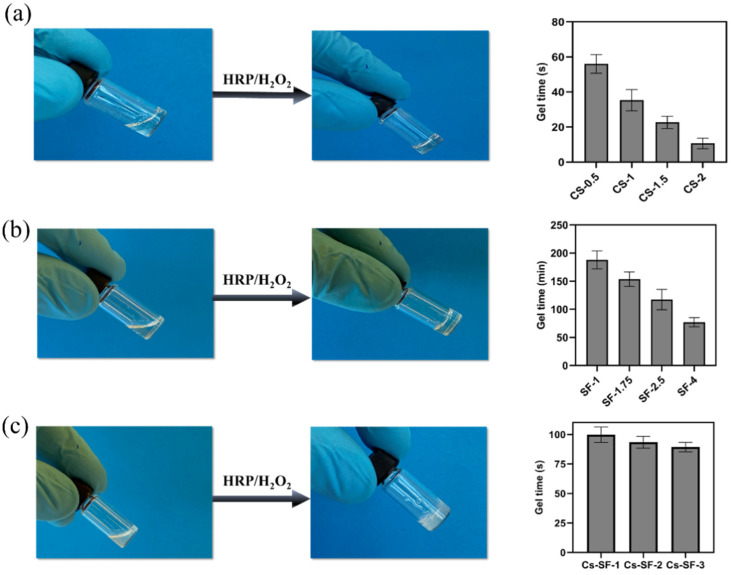
Pictures of sol–gel transition and gel time of the hydrogels: (**a**) Cs, (**b**) SF, (**c**) Cs-SF hydrogels.

A series of Cs, SF, and their mixture were prepared, and the gelation time was determined using the vial inversion method. Cs hydrogels with different concentrations (0.5%, 1%, 1.5%, and 2%) (Table 1) exhibited a gel time of 10–56 s, at a constant concentration of HRP (1 U/mL) and H_2_O_2_ (1 mM). The gelation time decreased from 56 to 10 s by increasing the Cs concentration from 0.5% to 2% (Figure 2a). Indeed, a high concentration of Cs (e.g., 2%) provides more phenol groups accelerating the gelation kinetic, due to the generation of more phenoxyl radicals in the catalytic cycle of HRP in the presence of H_2_O_2_. However, the short gelation time of Cs hydrogel can hinder its clinical application for potential drug and cell encapsulation [20], since the short gelation times can cause an inhomogeneous cell spreading inside the hydrogel and inconsistent cell responses [21].

The SF hydrogel was prepared similarly to the Cs hydrogel, at a constant concentration of HRP (1 U/mL) and H_2_O_2_ (1 mM), with different concentrations of SF (1%, 1.75%, 2.5%, and 4%) (Table 1). SF hydrogels showed a longer gelation time compared to the CS hydrogel. SF-1 exhibited a gelation time of more than 3 h. Increasing the SF concentration from 1% to 4% shortened the gelation time from 188 to 77 min, due to the higher content of tyrosine, which is still not suitable for biomedical application (Figure 2b). Long gelation time leads to producing soft gel for the in situ formation of hydrogel that is influenced by body fluid, and results in the decomposition of the hydrogel before stabilization [11].

Moreover, several studies reported that the long gelation time of SF hydrogel ranged from minutes to a few hours, depending on the SF, HRP, and H_2_O_2_ concentration used in the HRP-mediated crosslinking method. For example, Li et al. reported a gelation time of approximately 80 min for SF hydrogel (3%) at an HRP concentration of 10 U/mL. Alternatively, another study showed that increasing the SF concentration from 1 to 6% decreased the gelation time from 57.5 to 12.5 min at a constant concentration of HRP (10 U/mL) [22,23]. The higher gelation of the SF hydrogel in this study, compared to the reported studies, is due to the lower concentration of HRP (1 U/mL) used in our experiments. The HRP concentration has a significant effect on the gelation time. The gelation time decreases with the increase of the HRP concentration, due to the faster creation of the phenoxy radical in the coupling reaction [24].

The Cs-SF hydrogels were prepared by combining CS (1%) with SF of different concentrations (1%, 1.75%, and 2.5%), at a 1:1 volume ratio (Table 2).

In contrast to the Cs and SF gel precursor, the Cs-SF gel precursor showed turbidity, which could be due to the weak hydrogen bonding between hydroxyl and the amine groups of SF and Cs [25,26]. However, phase separation was not observed, and the Cs-SF gel precursor demonstrated an easy pipetting, indicating a consistent homogeneous mixture. The Cs-SF hydrogel gelation time was 99 s for Cs-SF-1 hydrogel and 89 for Cs-SF-3 (Figure 2c). The results showed that increasing the SF hydrogel did not significantly affect the gelation time. The gelation time of the Cs-SF hydrogel was dominated by chitosan, due to its short gelation time. The addition of Cs to SF hydrogel significantly decreased the gelation time, compared to the SF hydrogels alone.

### 2.3. Rheological Properties

**Figure 3:**
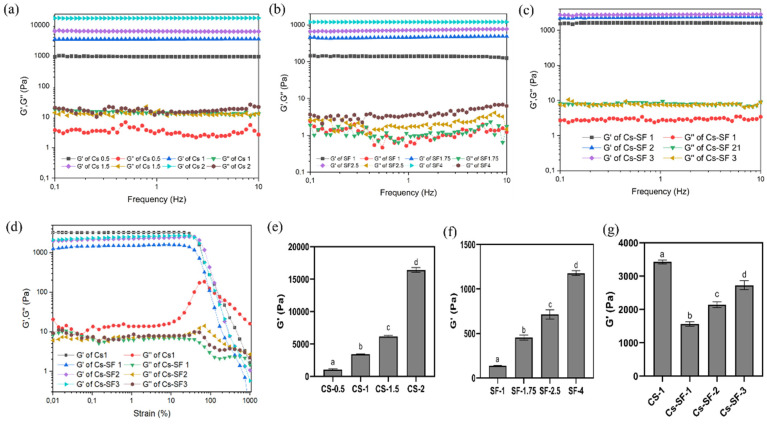
Rheological properties of chitosan (Cs), silk fibroin (SF), and Cs-SF hydrogels. (**a**–**c**) storage modulus (G’) and loss modulus (G”)—frequency dependence of Cs (**a**), SF (**b**), and Cs-SF (**c**) hydrogels at a constant strain of 1% at 37 °C; (**d**) storage modulus (G’) and loss modulus (G”)—strain dependence of Cs-1 and Cs-SF hydrogels at a constant frequency of 1 Hz at 37 °C; (**e**–**g**) average value of G′ of Cs (**e**), SF (**f**), and Cs-SF (**g**) hydrogels at 1 Hz. ^a–d^ bars that do not share a letter are significantly different at *p* < 0.05.

The rheological properties of Cs, SF, and Cs-SF hydrogels were investigated using an oscillatory test. The storage modulus (G’) and loss modulus (G”) of Cs hydrogels were evaluated using a frequency sweep test at a constant strain of 1% at 37 °C (Figure 3a).

All Cs hydrogels exhibited a frequency-independent G’ in the range of 1 to 10 Hz. A higher value of G’, compared to G”, demonstrates the elastic and rigid structure of the Cs hydrogels [27]. Moreover, Cs hydrogels exhibited a composition-dependent G’. The average G’ of Cs hydrogel increased from 1 kPa to 16 kPa at a frequency of 1 Hz, by increasing the Cs concentration from 0.5 to 2% (Figure 3e). The results revealed the effect of the Cs concentration on the stiffness and elastic properties of the Cs hydrogel, due to the increment in crosslinking density and the higher stiffness of the polysaccharide itself. However, the Cs hydrogels exhibited a damping factor (G”/G’) of between 0.0001 and 0.001, demonstrating the rigid and brittle structure of Cs hydrogel, due to the poly-β-(1,4)-D-glucosamine structure of Cs with high rigidity and its unsatisfactory energy dissipation mechanism [10]. The high G’ (3–16 kPa) of Cs hydrogels demonstrates that Cs with high rigidity can be used as a primary hydrogel network [28].

Similar to the Cs hydrogels, the G’ and G” of SF hydrogels were investigated using a frequency sweep test, at a constant strain of 1% at 37 °C (Figure 3b). The independence of SF hydrogels G’ to a frequency in the range of 1-10 Hz was observed, demonstrating the elastic behavior of the SF hydrogel. For example, SF-1 hydrogel exhibited an average G’ of 140 Pa, and the G’ increased to 1200 Pa by increasing the concentration to 4%. The results show that the SF hydrogels are weak in strength, which is consistent with the previous studies reporting the low mechanical strength of SF hydrogel identified by enzyme-mediated crosslinking [29,30]. A recent study reported a G’ of less than 100 Pa for 1% SF hydrogel HRP-mediated crosslinking, which increased to 750 Pa by increasing the SF concentration to 6%, demonstrating the low mechanical stability of SF hydrogel [23].

The damping factor of SF hydrogels was between 0.005 and 0.01, which was significantly higher than Cs hydrogel (0.0001–0.001), demonstrating the less elastic behavior compared to the Cs hydrogel. Hence, the results indicate that, unlike the Cs hydrogel, SF hydrogels exhibited less stiffness and rigidity. The addition of SF to the Cs hydrogel can increase the viscoelasticity of Cs hydrogels [31,32,33].

The frequency sweep analysis of Cs-SF hydrogels (Figure 3c) exhibited a similar trend compared to the Cs and SF hydrogel. All of the hydrogels exhibited a higher and frequency-independent G’, compared to the G”, in the range of 1 to 10 Hz, indicating the Cs-SF hydrogel’s elastic nature and ability to preserve the initial structure by increasing the frequency [34]. The results show that the addition of SF (1%) to CS (1%) decreased the G’ from 3500 Pa (Cs-1) to 1600 Pa (Cs-SF1) (Figure 3g), possibly due to the lower stiffness of SF compared to the Cs, as stated above. However, increasing the SF concentration to 2.5% improved the G’ of the Cs-SF hydrogels to 2800 Pa, which is still lower than the G’ of Cs-1 hydrogel.

Furthermore, an amplitude sweep test was performed to investigate the viscoelastic properties of the Cs-SF hydrogels (Figure 3d). This was applied to the Cs1 and Cs-SF hydrogels in the strain range of 0.1 to 1000%, at a constant frequency of 1 Hz and 37 °C. All of the hydrogels exhibited a strain-independent G’ up to 15% strain, demonstrating the linear viscoelastic region of the hydrogel, for which the G’ is independent of shear strain [35]. The hydrogels G’ gradually decreased after a strain of 15%, and crossed the G” that is attributed to a critical strain, correlated to the thixotropic feature [36]. The Cs-1 hydrogel exhibited a critical strain of 170%. In comparison, the critical strain of Cs-SF hydrogels was 415%, indicating that the addition of SF increased the strain resistance of the hydrogel, due to the improvement of the toughness and flexibility of the hydrogel. Indeed, similarly to the frequency sweep results, the addition of 1% SF decreased the G’ of the Cs-1 hydrogel to half of its value within the linear viscoelastic region, while increasing the hydrogel’s toughness and flexibility.

### 2.4. Microstructure, Water Uptake, and Degradation

**Figure 4:**
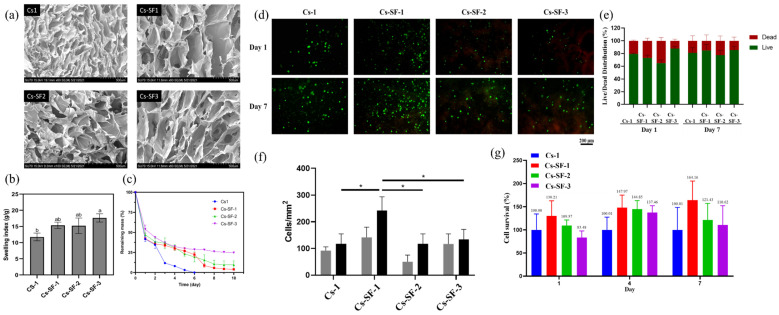
(**a**) Microporous structure of Cs-1, Cs-SF1, Cs-SF2, and Cs-SF3 hydrogels; (**b**) swelling index of freeze-dried hydrogels in PBS solution (10 mM) at 37 °C; (**c**) degradation behavior of Cs-1 and Cs-SF hydrogels in lysozyme solution at 37 °C—a, b bars that do not share a letter are significantly different at *p* < 0.05; (**d**) cell viability and proliferation fluorescence microscopic images of LIVE/DEAD staining of ovarian stromal cells encapsulated in Cs-1, Cs-SF-1, Cs-SF-2, and Cs-SF-3 on day 1 and 7—scale bar indicates 200 µm; (**e**) LIVE/DEAD distribution of ovarian stromal cells encapsulated in Cs-1, Cs-SF-1, Cs-SF-2, and Cs-SF-3 on day 1 and 7; (**f**) quantification of viable cells from LIVE/DEAD staining, using ImageJ software (https://imagej.nih.gov/ij/ (accessed on 1 September 2021))—asterisk marks (*) significant differences at *p* < 0.05; (**g**) graph showing cell survival obtained by PrestoBlue analysis on day 1, 4, and 7 (mean ± S.D.; *n* = 3).

Hydrogels’ network microstructure is one of the most important properties influencing the hydrogel’s ability of protein adsorption, cell proliferation, mass, and nutrition transfer [37]. Moreover, hydrogels with interconnected porose structures can regulate cell behavior by allowing cell adhesion, migration, and cell-cell interaction [38]. The microstructure of Cs-1 and Cs-SF hydrogels is shown in Figure 4a–d. All of the hydrogels exhibited a porous structure with pore sizes in the range of 50–350 µm. The addition of SF to Cs hydrogel increased the pore size of the hydrogel, possibly due to the decreasing crosslinking density, compared to the Cs-1 hydrogel with a denser microstructure [23]. Moreover, a significant difference between Cs-SF hydrogel’s morphology was not observed, and all of the hydrogels demonstrated a well-distributed porous structure, paving the way for the proliferation and migration of cells. The hydrogels demonstrated a heterogeneous microstructure with irregular pore shapes, un-uniformly distributed through the hydrogels, affected by the biopolymers’ structure, freeze-drying process (temperature, pressure), the water content of the hydrogel, as well as the possible interactions between Cs and SF [39,40].

The swelling index (SI) of the freeze-dried Cs-1 and Cs-SF hydrogels are shown in Figure 4b. The swelling index was determined through the immersion of the hydrogel in PBS solution (pH 7.4) at 37 °C. The swelling index is an essential feature of hydrogels for biomedical application, which depends on various factors, such as the crystalline/amorphous structure, crosslinking density, and, more importantly, the microporous structure and porosity of a hydrogel [41]. All of the hydrogels exhibited a high swelling index (>10), indicating the high porosity and “open-cell” pore features, confirming the interconnected microporous structure of the hydrogel. Moreover, the Cs-1 hydrogel exhibited the lowest SI (11.7) among the hydrogels, possibly due to the higher crosslinking density of the Cs-1 hydrogel with a dense microstructure. A recent study reported that the SI of Cs-based hydrogels was in the range of 16–12, depending on the temperature of the solution. At 37 °C, the hydrogel exhibited a SI of 12, while at 25 °C, the SI reached 6 [42]. Indeed, the addition of SF to Cs hydrogel increased the SI of the hydrogel, due to the decrease in the crosslinking density and increase in the pore size of the hydrogel. However, no significant difference was observed in the SI regarding the Cs-SF hydrogel with different SF concentrations.

Furthermore, the degradation behavior of the Cs-1 and Cs-SF hydrogels was evaluated using a gravimetric method, which involved monitoring the mass loss of the hydrogels in lysozyme solution (1 mg/mL) at 37 °C (Figure 4c). The Cs-1 hydrogel rapidly exhibited a weight loss of approximately 70% after 2 days, and continuously degraded until complete dissolution after 6 days. The degradation mechanism of the Cs hydrogel through lysozyme involves the breakage of the glycosidic bonds of the Cs, and the amide bonds of the grafted phenol groups. The hydrogel completely disappeared after reaching a critical value of glycosidic bonds and the amide bond breakage [43]. However, the Cs-SF hydrogel demonstrated a different degradation behavior. The Cs-SF hydrogels exhibited a similarly fast degradation behavior up to 2 days, although the degradation slowed down after the second day.

Indeed, the fast mass loss of the Cs-SF hydrogel during the early stages of degradation could be due to the amide bond breakage of the conjugated phenol groups into the Cs backbone, leading to the fracture of covalent bonding between the Cs and SF chain. The mass loss of the hydrogels reached a plateau after 7 days, with a remaining mass of 3.9%, 9.5%, and 25.6% for Cs-SF-1, Cs-SF-2, and Cs-SF-3, respectively. These results demonstrate that the addition of silk to the Cs hydrogel could improve the degradation behavior, increasing the hydrogel’s stability. Similarly, a recent study reported that the addition of 300% silk to a Cs 3D printed scaffold decreased the degradation rate up to 50% [32].

### 2.5. Ovarian Cell Encapsulation and Viability

Regarding the 3D cell encapsulation capability of the Cs-SF hydrogel, and the effect of hydrogels on cell viability and the proliferation of ovarian stromal cells, the cells were encapsulated into the hydrogels and cultured for 7 days. The proliferation and viability of the cells was evaluated by performing a LIVE/DEAD assay on day 1 and 7. As Figure 4e demonstrates, all of the hydrogels could preserve the viability of cells above approximately 70% on day 1 and 7. Figure 4g shows that there was an increase in the number of viable cells in all of the hydrogels on day 7, compared to day 1. This result demonstrates the ability of hydrogels to support cell survival and proliferation. Moreover, the Cs-SF1 hydrogel yielded a higher number of viable cells (Figure 4f), compared to the other hydrogels at day 1 and 7 (141.7 and 241.7 cells/mm^2^, respectively), demonstrating the positive effect of adding 1% SF to 1% Ch on cell viability and proliferation (vs. Cs, Cs-SF-2, and Cs-SF-3 on day 7 *p* < 0.05). Based on the mechanical evaluation of our hydrogels, Cs-SF1 had less shear modulus than the other hydrogels (G’ = 1600 pa), which is similar to the shear modulus of the normal ovary. Wang et al. [44] and Banerjee et al. [45] compared cell proliferation in hydrogels with different mechanical properties and demonstrated that cells proliferate more in hydrogels with less stiffness. Moreover, Nguyen et al. [46] and Shi et al. [47] reported an inverse relationship between cell proliferation and shear modulus.

This observation was confirmed using PrestoBlue analysis. The fluorescent values of hydrogels were detected on day 1, 4, and 7, which were normalized by using the mean of 1% Cs and the cell-free hydrogels as 100% and 0% viability, respectively. As Figure 4c demonstrates, the Cs-SF1 and Cs-SF2 hydrogels could improve cell proliferation compared to Cs-1 and Cs-SF3, at different time points, indicating the positive effect of silk on cell proliferation. On the other hand, comparing the silk-containing constructs on different days demonstrated that cell survival decreased by increasing the concentration of silk. Moreover, only the Cs-SF1 hydrogel exhibited an increase in cell survival over time (cell survival average: 130.21%, 147.97%, and 164.16% on days 1, 4, and 7, respectively). Therefore, the 1% SF could be a suitable concentration to add to 1% Cs. Zeng et al. [48] reported that, although Cs had a poor connection between non-uniform pores, using combinations of silk and Cs could enhance the structure of pores. Indeed, the morphology and size of pores could be altered by changing the volume ratio of Cs and SF. On the other hand, the uniformity and connectivity of the pores could affect cellular growth and behavior. Based on our results in the cell viability analyses, it seems that the Cs-SF1 hydrogel provided a better substrate for cell growth.

## 3. Conclusions

Cs-SF hydrogels with controllable gelation time and mechanical tunability were successfully developed, using the enzymatically crosslinking method for human ovarian stromal cells. The results showed that adding SF to CS hydrogel can solve the issue of the high rigidity of Cs hydrogel, due to the higher flexibility of SF chains compared to Cs. Moreover, by changing the SF concentration, we were able to demonstrate tunable properties, such as viscoelasticity, and could control the hydrogel’s degradation rate. Indeed, the Cs-SF hydrogels, particularly Cs-SF 1, exhibited superior support for the proliferation and viability of the human ovarian stromal cell, compared to the Cs hydrogel, possibly due to its higher viscoelasticity, mimicking the extracellular matrix (ECM). This new hydrogel could provide a potential microstructure for enclosing human ovarian stromal cells, by combining the toughness and flexibility of SF and the phenolic chemistry of Cs-SF, which can be a promising candidate for the encapsulation and regeneration of ovarian follicles.

## 4. Materials and Methods

### 4.1. Materials and Reagents

Chitosan with a minimum degree of deacetylation (DD) of 75%, sodium alginate, 1-Ethyl-3-(3dimethylaminopropyl) carbodiimide (EDC), N-Hydroxysuccinimide (NHS), horseradish peroxidase (HRP), sodium carbonate (Na_2_CO_3_)_,_ lithium bromide (LiBr), Hydrochloric acid 37% (HCl), sodium chloride (NaCl) and hydrogen peroxide (H_2_O_2_) (30%) were purchased from Sigma-Aldrich (St. Louis, MO, USA). Deuterium oxide (D_2_O) and trifluoroacetic acid-*d* (CF_3_COOD) were purchased from Eurisotop (Saint-Aubin, France). 3-(4-hydroxyphenyl) propionic acid (pHp) was purchased from Carbosynth (Compton, UK). 

### 4.2. Silk Fibroin Extraction

Silk fibroin solution was prepared in *B. mori* cocoons, using a previously described method [49]. Briefly, the sericin of the cocoons was separated from the fibroin by boiling 5 g of cocoons in 2 L Na_2_CO_3_ (0.02 M) for 60 min. Then, the SF fibers were washed three times with deionized water, to remove residual Na_2_CO_3_, and dried overnight at room temperature. The SF fibers were dissolved in 9.3 M LiBr at 60 °C for 4 h. The SF solution was then dialyzed using a dialysis membrane with a 3.5 kDa cut off against deionized water for 3 days, changing the water 3 times per day. After removing the LiBr, the SF solution was centrifuged 2 times at 9000 rpm, 5 °C, 20 min to remove the undissolved SF fibers. The concentration of SF solution was measured by weighing a dried sample of a known volume.

### 4.3. Chitosan–Phenol Conjugation

Phenol groups were conjugated with amine groups of Cs backbone using carbodiimide coupling chemistry, as described in [50], with some modification. Briefly, 1 g of Cs was dissolved in 100 mL of HCl aqueous solution with a pH adjusted to 5.5. Then, 1 g of 3-(4-Hydroxyphenyl) propionic acid (pHp) dissolved in 20 ethanol mixture (50% *v*/*v* DIW diluted ethanol) was added to the Cs solution, followed by adding EDC (26 mM) and NHS (26 mM). The pH of the solution was kept at 5.5 by the dropwise addition of 1 M HCl, and the mixture reacted for 24 h at 25 °C. The solution was dialyzed in distilled water (pH 4.5) containing 30 g of NaCl for 3 days, changing the water every 8 h, followed by dialyzing in distilled water for 4 h. The final product was freeze-dried and kept in a moisture-free desiccator before use. ^1^H NMR and Ultraviolet-visible analysis were used to monitor the phenol conjugation and determining the substitution degree.

### 4.4. Hydrogel Formation

A series of hydrogels based on Cs, SF, and their mixture were prepared in 1 mL vials at 37 °C, based on a method described in [51], with some modification. For the Cs and SF hydrogels, different concentrations of Cs-Ph (0.5%, 1%, 1.5%, and 2%) were prepared in PBS (pH 7.4) solution. SF solutions (0%, 1%, 1.75%, and 2.5%) were prepared by diluting from the degummed SF solution (5%) using deionized water. Then, the hydrogels were formed in a 1 mL vial tube. Briefly, 90 µL gel precursor containing 10 µL of HRP (10 U/mL) was mixed with 90 µL of gel precursor containing 10 µL of H_2_O_2_ (1 mM), and the gelation time was recorded using the tube inversion method.

Cs-SF dual network hydrogels were prepared by mixing Cs-Ph (1%) with different concentrations of SF solution (0%, 1%, 1.75%, and 2.5%) with a volume ratio of 1:1, and the hydrogels were formed the same way as above.

### 4.5. Physiochemical Characterization

The swelling index was determined using the method previously described [27]. The freeze-dried hydrogels were weighed (*W*_0_) and then immersed in 10 mM PBS solution (pH 7.4) at 37 °C for 36 h to reach the equilibrium swelling. Then, the swollen hydrogels were removed, and the excess water was removed using filter paper (*W*_1_). The swelling index was calculated by *W*_1_/*W*_0_.

The in-vitro degradation of the hydrogels was determined using the gravimetric method [52]. First, 300 µL of hydrogels were formed and weighed accurately (*W*_0_). Then, the hydrogels were immersed in PBS solution (pH 7.4, 0.01 M) containing 1 mg/mL of lysozyme at 37 °C. At different time intervals, the hydrogels were removed and weighed (*W*_1_) after removing the excess superficial water. Fresh media were added to samples after each time interval. The remaining mass of the samples was calculated by the (*W*_1_/*W*_0_) × 100%.

The microstructure of the hydrogels was evaluated by a scanning electron microscope (SEM) (Hitachi SU-70, Tokyo, Japan). A total of 600 μL of hydrogels was prepared, followed by lyophilization. The freeze-dried hydrogels were cross-sectioned and coated with gold before performing the experiment.

### 4.6. Rheological Properties

The rheological measurement of Cs, SF, and CS-SF hydrogels was performed using a rheometer (Anton Paar MRC 302, Graz, Austria) equipped with a plate–plate geometry (25 mm). A volume of 300 µL of hydrogels were, in situ, formed on the rheometer plate at 37 °C. The storage and loss modulus were determined using oscillatory tests. First, an amplitude sweep ranging from 1 to 1000% was performed at a constant frequency of 1 Hz, to determine the linear viscoelastic region (LVR). The frequency sweep test was performed at a frequency ranging from 0.1 to 50 Hz and a constant strain of 0.1%. To investigate gelation kinetics, a time sweep oscillatory test was performed at a constant frequency of 1 Hz and a strain of 0.1% (LVR). The viscosity of gel precursors was investigated using the flowability test over the range of shear rate (0.1–1000 s^−1^).

### 4.7. Cell Isolation and Culture

Ovarian biopsies were taken from menopausal patients undergoing gynecologic surgeries. The use of human ovaries was approved by the Institutional Review Board of the Université Catholique de Louvain on May 2019 (IRB reference 2012/23MAR/125). The ovaries were frozen, based on the Gallardo et al. protocol [53]. After tissue thawing, stromal cells were isolated, as previously described by Asiabi et al. [54]. Briefly, the tissue was minced using a tissue chopper (Campden Instruments LTD, London, UK), then transferred in DPBS with Ca^2+^ and Mg^2+^ (14040-091; Gibco, Paisely, UK) containing 0.28 Wünsch units/mL Liberase DH (05401089001; Sigma-Aldrich, Mannheim, Germany) and 8 Kunitz units/mL DNase (89836; Thermo Scientific, Vilnius, Lithuania). Enzymatic digestion was performed at 37 °C under 150 rpm agitation, with pipetting every 15 min. After 75 min, the inactivated solution containing DPBS without Ca^2+^ and Mg^2+^ (10010-015; Gibco, Paisely, UK) and heat-inactivated fetal bovine serum (HI FBS; 16140071; Gibco, Paisely, UK) was added to the digested cell suspension. After passing through 80 μm (NY8002500; Millipore, Darmstadt, Germany) and 30 μm (NY3002500; Millipore, Darmstadt, Germany) nylon net filters, the suspension was centrifuged at 500× *g* for 10 min. After counting with trypan blue (T8154; Sigma-Aldrich, Darmstadt, Germany), the cells were cultured in DMEM/F12 1X (21041-025; Gibco, Paisely, UK), supplemented with 10% HI FBS and 1% antibiotics and antimycotic (15240-062; Gibco, Green Island, NY, USA), at 37 °C, 5% CO_2_ in a humidified atmosphere. Passage 6–8 cells were used to encapsulate the cell suspension in the hydrogels.

### 4.8. 3D Cell Encapsulation

After washing the cells with PBS, the cells were detached using Accutase (A6964; Sigma-Aldrich, Sigma-Aldrich, Darmstadt, Germany), centrifuged, and resuspended in the hydrogel precursors (Cs-1, Cs-SF1, Cs-SF2, Cs-SF3) containing HRP (1 U/mL), to achieve the cell density of 8 × 10^5^ cells/mL. Equal amounts (15 µL) of gel precursors containing cells and HRP were subsequently mixed with the same gel precursors containing 1 mM H_2_O_2_, and incubated at 37 °C for 25 min to form a hydrogel. To avoid hypoxia, we used a low volume of hydrogel (30 µL) in the form of droplets that did not cover the complete surface of the wells. The plates were not shaken and precursors were mixed only by pipetting [55,56]. Then, 300 µL of culture medium was added to the hydrogels in the 48-well plate and cultured at 37 °C, 5% CO_2_ in a humidified atmosphere. The medium was changed every other day.

### 4.9. Mitochondrial Activity

The cell-encapsulated hydrogels were prepared in triplicates, and after 1, 4, and 7 days of in-vitro culture, cell mitochondrial activity was assessed using PrestoBlue^TM^ HS cell viability reagent (P50200; Invitrogen, Invitrogen, Oregon, USA), according to the manufacturer and Gu et al. [57] protocols. Briefly, PrestoBlue solution was prepared at a concentration of 1:10 in culture medium, and after removing culture medium, it was added to the wells containing cell-encapsulated and cell-free hydrogels, and incubated at 37 °C for 3 h. Subsequently, 100 µL PrestoBlue solution in each well was transferred onto a 96-well plate. The absorbance was read at the emission wavelength of 620 nm and the excitation wavelength of 560 nm using fluorescence spectroscopy (Multilabel reader, Victor X4, Perkin Elmer, Singapore Singapore). The experiment was performed in 3 independent replicates.

### 4.10. Cell Viability (LIVE/DEAD)

The survival of cells encapsulated in hydrogels was evaluated using the LIVE/DEAD™ viability/cytotoxicity kit (L3224; Invitrogen, Oregon, USA), based on Amorim et al.’s protocol . The hydrogels on day 1 and 7 were washed with warm DPBS and incubated with the mixture of ethidium homodimer-1 and calcein-AM in DPBS at 37 °C for 45 min. After washing the hydrogels with DPBS, the live and dead cells (green and red colors, respectively) were visualized through a fluorescent microscope (Leica, Wetzlar, Germanyusing two different filters (ex/em 495/515 nm and ex/em 495/635 nm).

## Figures and Tables

**Table 1:** Parameters and gelation time of chitosan (Cs) and silk (SF) hydrogels.

Sample Name	CS-Ph Concentration (wt%)	SF Concentration (wt%)	Gelation Time
CS-0.5	0.5	0	56 ± 4 s
CS-1	1	0	35 ± 4 s
CS-1.5	1.5	0	22 ± 2 s
CS-2	2	0	10 ± 2 s
SF-1	0	1	188 ± 13 min
SF-1.75	0	1.75	153 ± 10 min
SF-2.5	0	2.5	117 ± 14 min
SF-4	0	4	77 ± 6 min

**Table 2:** Parameters and gelation time of Cs-SF hydrogels.

Sample Name	Mass Ratio of Cs to SF	Cs-Ph Concentration (wt%)	SF Concentration (wt%)	Gelation Time (s)
Cs-SF-1	1:1	1	1	99 ± 5
Cs-SF-2	0.57:1	1	1.75	93 ± 5
Cs-SF-3	0.4:1	1	2.5	89 ± 4
